# Improving the Quality of Maternity Care through the Introduction of Professional Midwives and Mentoring in Selected Sub-District Hospitals in Bangladesh: A Mixed Method Study Protocol

**DOI:** 10.3390/mps5050084

**Published:** 2022-10-21

**Authors:** Rondi Anderson, Sojib Bin Zaman

**Affiliations:** 1 Faculty of Health and Medicine, Lancaster University, Lancaster LA1 4YW, UK; 2 Department of Medicine, Monash University, Melbourne 3800, Australia

**Keywords:** midwives, midwifery-led care, mentoring, maternity care, rural Bangladesh

## Abstract

**Introduction:** Bangladesh introduced professional midwives in 2018 to address gaps in sexual and reproductive health services, focusing on improved maternity care. Facility mentoring has been introduced in selected facilities within the government to enable midwives as they move into their new roles. **Objectives:** To describe a protocol (1) to determine if introducing international standard midwives in rural sub-district hospitals in Bangladesh, both with and without facility mentoring, improve the availability and quality of maternal and newborn health care compared to the facility without midwives; and (2) to explore the experiences of the midwives, and the maternity staff and managers that they joined, following their introduction. **Methods:** This will be a mixed-methods study to examine differences between selected hospitals grouped into three categories: without midwives (only nurses), with midwives, and both with midwives and mentorship. Hospital selection will be based on choosing those with the highest birth caseload. The quantitative component will consist of facility observations and clinical data extraction to assess their (hospital and midwives) readiness (birth preparedness and complication readiness) and clinical care to explore whether facilities with newly introduced midwives have improved availability and quality of care. We will use facility assessment tools to extract clinical data. In addition, we will use a structured open-ended interview guideline to conduct focus groups and in-depth interviews to understand the perceptions, attitudes, and experiences among maternity staff (e.g., nurses and paramedics) and health managers (e.g., facility manager, residential medical officer, consultants), as well as the midwives themselves toward the newly introduced midwives and the quality of care. We plan to use a fixed effect logistic regression to compare the relationship between variables in the three hospital types for each observed data point. For analyzing qualitative data, we will adopt content analysis and use NVivo to identify themes related to perceptions, attitudes, and experiences. **Expected results:** The introduction of professional midwives may improve the quality of maternal health care in rural settings. The addition of a mentoring program can support midwives in transitioning into their new roles and introduce improved care quality.

## 1. Introduction

### 1.1. Background

Despite decades of global prioritization, pregnancy-related morbidity and mortality remain a significant public health and human rights concern for the world’s poorest such as low-and-middle-income countries (LMICs) [1,2]. Being an LMIC, Bangladesh is not an exception. Although Bangladesh has improved in many social indicators in the last 20 years, it still lags in critical areas, including maternal health and gender equity. For example, Bangladesh successfully reduced the maternal mortality ratio (MMR) from 500/100,000 in 1980 to the current rate of just under 173/100,000 (2017). However, since 2010 there has been very slow decline in MMR, and the Millennium Development Goals for maternal mortality were not met [3]. The poorest women in Bangladesh are ten times more likely to die from pregnancy-related complications than the wealthiest women [4].

Bangladesh also faces a critical shortage of health workers to provide quality maternity care. In 2018 there were 83 doctors, nurses, and midwives per 100,000 population [5] against the recommended threshold of 44.5 health workers per 10,000 population [6]. In addition, health workers are less concentrated in rural areas where most of the population still resides [7]. However, vacancies and absenteeism of service providers, coupled with poor infrastructure for maternal and neonatal health services in hard-to-reach areas, place a burden on the system to render timely and quality services [8]. The Government of Bangladesh (GOB) has developed a new cadre of diploma midwives to improve sexual and reproductive health (SRH) services in response to these identified gaps. In addition, the Ministry of Health and Family Welfare (MOHFW) endorsed a midwifery strategy, job description, scope of practice, and orientations for health facility managers to help them understand midwifes’ roles as per World Health Organization (WHO) quality standards [9]. 

As the profession of midwifery—distinct from nursing—is new in Bangladesh, and poor-quality routines persist in maternity care areas, midwives may need some initial support from mentors to step into their new roles and help maternity care transition to improved quality. Until the deployment of midwives in 2018, nurses had been considered ‘midwives’ in addition to their nursing role in Bangladesh [10]. As nurses do not meet a global standard for midwives [11], they are now being shifted to work in their areas of expertise. Midwifery education and regulations are set according to the International Confederation of Midwives (ICM) recommendation, and midwives’ expertise is in SRH areas, including maternity units [12]. However, there is resistance to midwives taking leadership in SRH as the profession, and many of the WHO-guided methods of care are new in Bangladesh [13]. Therefore, with the support of a non-governmental organization (NGO), Save the Children, the GOB has implemented a mentorship program in 2017 for selected facilities to support the facility managers (e.g., by providing educators, training, and equipment) to enable the midwives to perform their duties. However, there are some public hospitals where midwives have not been deployed. To date, nurses perform midwifery roles in addition to their regular nursing duties in the absence of midwives. Hence, it is an opportunity to evaluate the effectiveness of midwives with mentorship in providing WHO quality of care.

### 1.2. Rational

The introduction of professional midwives is linked to increased utilization of maternal health services and quality of care [14,15]. In addition, mentoring and supervision can improve health worker performance and provide enabling environments to strengthen health systems [16,17]. Deussom et al. conducted a recent systematic review (2022) and indicated a direct impact of clinical mentoring or supervision on the improved performance of health workers [17]. The research conducted in Rwanda, India, Nepal, Ethiopia, South Africa, and Uganda found that mentoring does improve quality. More often than not, the impact that mentoring makes is selective, and gaps remain even when improvements are made [16,18,19,20]. Therefore, it requires an investigation of the impact of ICM-standard midwives and the benefits of mentoring during the initial deployment phase of midwives. However, we are unaware of any article that has explicitly looked at the impact of mentoring on a recently introduced professional cadre in LMICs. The present paper describes a study protocol to evaluate if introducing ICM-standard midwives, both with and without mentoring, improves the availability and quality of maternity care in rural sub-district hospitals in Bangladesh. We also describe a framework that can be used to assess potential barriers and facilitators from the experiences of midwives and maternity staff (e.g., nurses and paramedics) in providing quality midwifery care. We prepared this manuscript in accordance with the SPIRIT reporting checklist (Supplementary File S1).

### 1.3. Research Questions

- Is there an association between newly introduced professional midwives, with and without mentoring, and improved availability and quality of maternal and newborn health care in sub-district hospitals in Bangladesh?
- What are the experiences of the midwives, the maternity staff, and health managers (e.g., facility manager, residential medical officer, consultants), in establishing midwifery care?

### 1.4. Hypothesis

There is a positive impact on the introduction of ICM-standard midwives and the benefits of mentoring during the initial deployment phase. In addition, perceptions of midwives’ capabilities, both among midwives themselves and the other maternity staff they worked with, improve with mentoring.

### 1.5. Objectives

(1) To determine if a national deployment of midwives in a low-resource setting improves evidence-based maternal and newborn care practices with and without facility mentorship.
(2) To explore the experiences of midwives providing care, as well as non-midwife maternity staff and health managers, to understand the underlying motivators that facilitated and challenged the introduction of midwives.

## 2. Experimental Design

### 2.1. Study Design and Setting

We have chosen a pragmatic mixed-methods approach with a systems-thinking theoretical framework for this study. This framework includes a wide range of qualitative and quantitative methods and tools designed to understand better system behaviors and interventions in complex situations [21]. Furthermore, as the research will be a natural experiment, a pragmatic approach can facilitate focus on research questions in a real-life context [22]. 

Government sub-district hospitals will be selected that performed the most births and met the criteria for the three groups of hospitals—(i) with no midwives; (ii) with at least four midwives without facility mentoring support; and (iii) with at least four midwives with facility mentoring. Each hospital will be visited once to collect data, and the results will be compared and contrasted to assess differences across the hospitals. The quantitative and qualitative data will be collected simultaneously during hospital visits. Two types of quantitative data collection approaches—survey and observation—and two types of qualitative methods—interview and focus group discussions—will be used for triangulation [23]. The qualitative data will shed light on the clinical observations and survey results in the different hospital groups. 

### 2.2. Theoretical Framework 

We will use the systems-thinking approach as a theoretical framework (Table 1). As per WHO, it can be defined “systems Thinking for Health Systems Strengthening offers a practical approach to strengthening health systems through a ‘systems thinking’ lens” [24]. Systems thinking approach can enable stakeholders to understand complex situations by observing the whole scenarios, patterns, and interrelationships on health problems rather than focusing on system parts separately [21]. This framework has recently been used as a model to address the complex health systems in LMICs [2]. Systems thinking highlights that complex systems contain many sub-systems within a more extensive umbrella system. However, some sub-systems do not align and may dominate because of limited vision or self-interest from the researcher’s perspective. This bias can cause sub-optimization of an intervention across the entire system and impede the attainment of the primary objective [25,26,27]. The system thinking approach allows researchers to identify the root causes of public health challenges and think of new opportunities to progress further. It also considers various stakeholders’ views, interests, and power dynamics when evaluating complex public health interventions [28,29].

### 2.3. Interventions

#### 2.3.1. The Midwifery-Led Continuum of Care

Introducing midwives into the public sub-district hospitals is intended to improve the availability, quality, acceptability, and accessibility of facility ANC, birth, and appropriate response to obstetric emergencies. In addition, they are able to provide women with pre-conception counselling and family planning.

#### 2.3.2. Health Facility Mentoring 

Health facility mentoring is defined as providing mentoring support for the health managers who support individual providers and the facility systems that support interventions [30,31,32]. For this research, the mentorship will be conducted by mentors who are graduated female doctors. This choice of female doctors is justified for two reasons: (i) the lack of senior midwives; and (ii) the need for mentors to have authority within the hierarchical hospital system. Mentors will receive a 1-week orientation on the role of midwives, guidelines on the *WHO standards for improving the quality of maternal and newborn care in facilities*, and 2–3 days of field site hands-on experiences. This mentorship will be scheduled to have fortnightly visits to a hospital to guide and provide hands-on support to the midwives to establish evidence-based midwifery care as per ICM standards. They will also advocate with health managers to create an enabling environment for the midwives to establish quality maternity care. 

### 2.4. Eligibility Criteria of the Hospital 

We will select sub-district hospitals that are mainly homogeneous. Selection will be made purposively by identifying the sub-district hospitals that performed the most births in the previous six months. We will consider more than 600 births to be delivered over the last six months in a selected facility. We will invite 24 sub-district hospitals to participate in our evaluation and expect to include at least 18 hospitals from each hospital group. Four midwives needed to provide care, as per the government recommendation, to meet the criteria of having midwives. Each sub-district hospital is required to meet the criteria for one of the three groups—no midwives, at least four midwives without facility mentoring, and at least four midwives with facility mentoring. 

### 2.5. Team of the Data Collectors

We will recruit a data collection team consisting of the primary researcher, a facility organizer, two research assistant midwives, and a translator. They will be trained on various data collection tools by the investigators. The data collector team will visit one selected hospital once to collect both the qualitative and quantitative data concurrently. In addition, we will recruit a national facility organizer who will work as a peer with the hospital managers to organize the focus groups and interviews. The primary investigator and translators will conduct interviews and focus group discussions. At the same time, research assistant midwives will observe women in labour (in-patient department) and women receiving ANC (outpatient department). The research assistant midwives will also administer the survey questionnaires to the selected maternity and emergency staff working in the hospital. 

### 2.6. Ethical Approval

Ethics approval has been obtained from the Faculty of Health and Medicine Research Lancaster University Ethics Approval Committee. In addition, ethical approval has also been taken from the Centre for Injury Prevention and Research Bangladesh (CIPRB) to perform the research in Bangladesh. 

## 3. Materials and Equipment

### 3.1. Data Collection Tools

Data collection will include structured observations, extraction of information on service utilization from register books, survey responses, and qualitative focus groups and in-depth interviews. We will use different methods to explore the impact and experiences of deploying midwives (with and without mentoring) for maternity care. The first two data collection methods will answer the first research question concerning improved availability and quality of maternal and newborn care. The survey method will also explore perceptions and experiences. Two observation tools will consist of direct observations of clinical services and extraction of service utilization data from the service registers. In addition, we will collect service utilization data of births and observation of WHO quality indicators to assess and quality of maternity services with or without the deployment of midwives and the influence of mentoring. 

### 3.2. Field Testing of the Data Collection Tools

The three quantitative data collection tools examining evidence-based maternity care practices are (1) hospital readiness, (2) clinical observations, and (3) a survey (Table 2). The survey questionnaire has been developed from an existing evidence-based practice survey tool used in Bangladesh [33]. We will administer the survey questionnaire to all consenting emergency and maternity staff and facility managers to explore the perceived knowledge, capacity use, and value of evidence-based maternal and newborn healthcare interventions. The survey questionnaire is prepared in English and then translated into Bengali by a professional translator. Back translation was done by a second professional translator and checked for construct validity. 

All data collection tools will be piloted in a sub-district hospital identical to those hospitals selected in the study. The tools will be evaluated and modified if required during and after the pilot visit. In addition, we will use a semi-structured interview guide to conduct focus group discussions and in-depth interviews. 

### 3.3. Operational Definition

#### 3.3.1. Professional Midwives 

As defined by the ICM, a professional midwife has a minimum pre-service education of a 3-year diploma focused on midwifery care that meets a standard ICM competency. She is an SRH health practitioner who employs the promotion of normal physiology supported by utilizing modern science and technology [34,35]. Table 3 provides a summary of critical concepts for the profession of midwifery as defined by the ICM [36].

#### 3.3.2. Health Facility Mentoring

Health facility mentoring is defined in this research as providing mentoring for individual providers and the facility systems that support implementing midwifery interventions [30,31,32].

### 3.4. Sample Size

#### 3.4.1. Quantitative Component 

We will use a convenient sampling for evaluating the quantitative component of this study. The sample size for the quantitative portion will be determined through power analysis to detect significant differences in implementing the observed WHO quality care interventions between three groups of hospitals. Using an alpha of 0.05 and beta of 0.8, a total sample size of 159 observations is recommended to detect a medium-size effect (f = 0.25). This 159-sample size will include all consenting emergency and maternity staff who conduct delivery, as well as all pregnant and immediate postpartum mothers receiving care during the observations. 

Based on some gray literature and informal observation from the researchers’ prior visits to hospitals of the same type, we have estimated that the proportion of the selected quality of care indicators being implemented would be less than 5% in the no midwife group, 20% in the midwives without mentoring group, and 50% in the midwives and mentoring group [31,37,38]. Using these estimated percentages with an alpha of 0.05 and a beta of 0.8, the recommended sample size per group for each comparison will be n = 60 for the comparison between the ‘no midwives’ and ‘midwives without mentoring’ groups, n = 12 for the comparison between the ‘no midwives’ and ‘midwives and mentoring’ groups, and n = 31 for the comparison between the ‘midwives without mentoring’ and ‘midwives and mentoring’ groups. We will approach 160 maternity staff to complete the survey (i.e., evidence-based practice survey) on their knowledge, perceptions, and use of the maternity care interventions.

#### 3.4.2. Qualitative Component

We will use purposive sampling to select the participants for focus groups and interviews. We are not able to decide in advance the number of respondents we will interview or how many FGDs to perform. The exact number will be determined when we are to achieve data saturation. However, we estimate to conduct 4–5 focus group discussions with midwives and maternity staff from each type of facility. We will choose 6–8 participants for conducting each focus group discussion, which is recommended as a realist approach to developing primary themes [39,40]. We will ensure that the participants have specific experiences and backgrounds as a prerequisite for participation.

### 3.5. Data Storage and Management

We will scan and store all handwritten field notes on a password-protected encrypted computer. Interviews recorded on an electronic device will similarly be stored in a password-protected computer. Quantitative data will be imported into a Microsoft Excel database. We will use the R statistical package to analyze quantitative data and NVivo to synthesize qualitative interviews. Observations and survey data will be transcribed electronically. These data will be backed up on the Lancaster University server in a password-protected file. Paper data will be kept in a locked cabinet and destroyed after ten years. 

### 3.6. Data Analysis Plan

#### 3.6.1. Quantitative Analysis 

Descriptive statistics (such as mean, median, mode, and variance) will be calculated to analyze the survey data. We will either use a log transformation or remove the outliers for the skewed distribution to convert it to a normal distribution. The analysis will examine the differences between hospital types and the type of respondent (i.e., midwives, nurses, and doctors). We will use cross-tabulations for binary questions to achieve proportions. Frequency totals will be reported and stratified by mentored and non-mentored groups. Tables and graphs will be used to report hospital readiness data. Service utilization trends showing differences between hospital types over the previous six months will be depicted in a line chart. Frequencies and proportions will be generated based on the clinical observation data. We will use a fixed-effect logistic regression test to compare the relationship between variables in the three hospital types for each observed data point. This test may delineate significant differences between the three groups’ use of defined maternal health practices. 

#### 3.6.2. Qualitative Analysis 

We will use the inductive method to analyze qualitative data and the systems thinking approach to highlight themes and salient points [41,42]. Data from focus groups and interviews will be transcribed. For analyzing qualitative data, we will adopt content analysis and use NVivo to identify themes related to perceptions, attitudes, and experiences. Content analysis is a method of listening for a sense of the whole rather than fracturing data into pieces to report focus groups and interviews [43,44]. Data from the survey will be analyzed separately to capture topic areas for coding to facilitate emerging themes derived from the WHO’s Health System Building Blocks approach [45].

## 4. Detailed Procedure

### 4.1. Procedure to Recruit Participants 

Two to four weeks before the data collection, a trained research assistant will visit the facilities, display posters, and circulate flyers announcing the research in the emergency and maternity wards and the ANC clinic. This information will make the hospital staff aware of the upcoming study and reach as many pregnant women as possible. Following the researcher’s visit, ANC staff should verbally inform all pregnant women about the forthcoming research, as literacy is limited for many women accessing public facilities [46]. Recruitment will be performed on the first day of the hospital visit through trained research assistants. 

### 4.2. Data Sources/Measurements

The hospital readiness data will be sourced from register books and direct observations of the management of clinical emergencies in the facilities. Data collectors will extract relevant data from the register. In addition, we will collect binary observational data for the clinical observations. For example, the measurement will be ‘yes’ or ‘no’. Here, ‘yes’ will denote the use of selected evidence-based care interventions or the presence of an aspect of facility readiness. Contrarily, a ‘no’ response will imply a lack of use or absence of a particular clinical practice. Observations will be made at unannounced times to reduce the risk of the Hawthorne effect [47,48,49]. The length of time spent conducting observations at each hospital will range from 5–10 days and will be depended on ensuring that minimum 10 births will be observed. The survey will take ~15–20 min to administer. Responses to quantitative questions will be combined into the binary (i.e., categorical) and Likert scale (i.e., ordinal). 

We will select participants for the focus groups and interviews via invitations to maternity staff and health managers available during the site visits. All conversations will be held in a private space in the hospital and recorded and transcribed verbatim with consent. Field notes will be completed at the end of each day. During the focus groups, the invited participants will discuss their perceptions, use of, and familiarity with evidence-based maternal and newborn health care. Where relevant, we will explore the facility mentoring experiences. Both interviews and focus group discussions will include staff and managers from three categories of hospitals- (a) hospitals with no midwives, (b) those with midwives but without mentoring, and (c) those with midwives and mentoring. For the focus groups, the non-midwife maternity staff will be separated from the midwives during the process. In addition, we will take interviews with managers, nursing supervisors, hospital head managers, obstetricians and gynaecologists, and medical doctors. Table 4 provides a summary of the qualitative data collection methods and respondents.

## 5. Expected Results

Midwives have gained separate and distinct-set of expertise in providing midwifery care, which has been documented in high-resource countries. Thus, introducing professional midwives may bring some good changes in rural sub-district hospitals compared to nurses. The addition of a mentoring program can support midwives in transitioning into their new roles and introduce improved care quality. Moreover, mentors can strengthen the capacity of midwives and nurses regarding new clinical practices and help to create an enabling environment for maternal care. Like other countries in LMICs, Bangladesh has recently introduced a globally standard midwife cadre into its health system. In addition, midwives are deployed to selected rural hospitals supported by a mentoring program to facilitate an enabling environment at the facility level for midwives to practice. This research will look at the effects of these interventions. However, our survey is based on self-report and this can raise social desired bias. We will adequately train and re-train our data collectors to be careful on it. 

A system-thinking theoretical framework will provide a structured lens for evaluating complex systems and determining the change within them. For example, we will be able to assess whether introducing an international standard midwifery cadre with or without mentorship in rural hospitals increase the evidence-based care practices as guided by WHO quality maternity care standards. This research will also enable us to explore barriers and facilitators related to midwives transitioning into their new roles in the sub-district hospital. The results of this project will be disseminated in academic journals, and research briefs will be used to aid the development of policy briefs. We also plan to advocate government to take the ownership of this mentorship program and initiate a pilot project in a small scale. 

## Figures and Tables

**Table 1:** Systems thinking evaluation questions for context evaluations *.

Contexts Evaluation
*What facilitates/impedes the intervention?* Motivations to provide quality care versus personal gain, enabling environments, supportive co-workers and managers, autonomy/agency of midwives
*What other co-interventions are relevant?* Supportive policies and guidelines, relevant training
*What else is changing in the system?* Momentum around health system improvement and reducing maternal mortality Increasing inequitable access to health care Resources shifting to urban health systems

* Adapted from: De Savigny, Don, and Taghreed Adam, eds. Systems thinking for health systems strengthening. World Health Organization (2009).

**Table 2:** Quantitative data collection forms (variables will be defined using a standard definition, as per WHO, to measure the criteria).

**Hospital Readiness Form**	
**Hospital readiness**	*Purpose:* Assess the readiness of hospitals to provide basic maternity care, including emergency response *Data sources:* Observation checklist and hospital register books *Variables (will be defined as per WHO/standard criteria’s):* Oxytocin in emergency & delivery rooms * Magnesium sulfate in emergency & delivery rooms* Newborn resuscitation area with Ambu bag in the delivery room* Separate ANC corner Diploma midwife staffing ANC corner Midwives staffing maternity area Register book with midwife identification used for births Register book for PPH and eclampsia* Number of births performed by midwives in the last 6 months Number of PPH cases in last 6 months Number of eclampsia cases in last 6 months * Indicates emergency preparedness variable *Analysis:* Descriptive statistics will be generated using frequencies and proportions
**Direct Observation Form**	
**Clinical observations**	*Purpose:* Direct observation of clinical practice *Data sources:* Observation Checklist *Variables (will be defined as per WHO/standard criteria’s):* Individual patient ANC cards used Skin-to-skin contact for 1 h Companionship in labor & delivery Partograph used during labor Upright position for labor & delivery Delayed cord clamping Active management of the third stage of labor *Analysis:* A series of analyses will be performed to determine statistical significance.
**Survey Form**	
**Evidence-based practice survey**	*Purpose:* Assess provider comfort with and use of evidence-based interventions *Data sources:* Provider self-reports, shared verbally in response to question prompts *Variables (will be defined as per WHO/standard criteria’s):* Importance of ANC Yes/No questions on whether the provider felt capable of and carried out these interventions: Partograph use Skin-to-skin contact for 1 h Initial care for PPH Initial care for eclampsia Likert scale questions asking for respondents’ opinions about the importance and or value of these interventions: Companion in labor and delivery Non-supine positioning for labor and delivery Diploma midwives providing maternity care True/False checkboxes: Whether the facility had recently changed in terms of performing ten critical evidence-based interventions If True, whether the introduction of mentors and separately midwives precipitated the change *Analysis:* Descriptive statistics will be generated using frequencies and proportions.

**Table 3:** Key concepts of the profession of midwifery by the International Confederation of Midwives (ICM).

Number	Concept
1	Respect for human dignity and women as persons with full human rights
2	Advocacy for women so that their voices are heard and their healthcare choices are respected
3	Cultural sensitivity, including working with women and healthcare providers to overcome those cultural practices that harm women and babies
4	A focus on health promotion and disease prevention that views pregnancy as a normal life event
5	Advocacy for normal physiologic labor and birth to enhance the best outcomes for mothers and infants
6	Autonomous when caring for healthy women, able to be the first responder in obstetric and newborn emergencies, and work in interdisciplinary teams when complications arise.

**Table 4:** Qualitative data collection methods and respondents.

Methods	Number of Respondents
Interviews (estimated number)	10 Nursing supervisors Five hospital head managers Three obstetrician-gynaecologist doctors Four medical doctors
Focus group discussions (FGD)	Approximately 6–8 respondents will participate in an FGD. We will conduct the following number of FGDs: One at hospitals with no midwives Two at hospitals with midwives but no mentoring Three at hospitals with midwives and mentoring

## Data Availability

Data sharing does not apply to this article.

## Supplementary Materials

The following supporting information can be downloaded at: https://www.mdpi.com/article/10.3390/mps5050084/s1, Supplementary File S1: Spirit checklist.
